# Maxpro Designs for Experiments with Multiple Types of Branching and Nested Factors

**DOI:** 10.3390/e26100856

**Published:** 2024-10-10

**Authors:** Feng Yang, Zheng Zhou

**Affiliations:** 1 School of Mathematical Sciences, Sichuan Normal University, Chengdu 610066, China; yangfeng@sicnu.edu.cn; 2 School of Mathematics, Statistics and Mechanics, Beijing University of Technology, Beijing 100124, China

**Keywords:** maximum projection, branching and nested, space-filling, criterion

## Abstract

Contemporary experiments often involve special factors known as branching factors. The levels of such factors determine the presence of some certain factors, referred to as nested factors. The design criteria for investigating the goodness of such designs are rarely developed. Furthermore, the existing criteria for such designs pay less attention to the space-filling property of low-dimensional projections of the design. The efficiencies of designs yielded by such criteria can markedly decrease when only a few factors are significant. To address this issue, this paper proposes a novel space-filling criterion based on the maximum projection criterion to evaluate the performance of the designs with branching and nested factors. A framework to construct optimal designs under the proposed criterion is also provided. Compared with the existing works, the resulting designs have better space-filling properties in all possible low-dimensional projections. Moreover, our strategy imposes no constraints on run size, level, and type of any factor, demonstrating its broad applicability.

## 1. Introduction

With the advancement of technology, the structures of experimental factors have become more and more complex. In many experiments, the presence of some factors is determined by the levels of specific factors, where the former factors are called nested factors and the latter factors are called branching factors. The remaining factors, apart from these two kinds, are referred to as shared factors. Here, we revisit the example in [1] to briefly illustrate these three kinds of factors. An experiment aims to optimize the turning process hardened for bearing steel. The factors of the experiment include cutting edge shape, cutting speed, cutting depth, and so on. Hone and chamfer are two commonly used cutting edge shapes, where chamfer length and chamfer angle can be altered when using a chamfered edge. In this experiment, the cutting edge shape is a branching factor, chamfer length and chamfer angle are two nested factors, and the other factors are shared factors. For more examples, see [2,3,4,5].

Space-filling designs are commonly used in situations where the relationship between factors and responses is a black box. The core idea behind such designs is to distribute design points evenly across the design space [6,7]. This facilitates a comprehensive exploration of the design space and guarantees that no area remains under-explored. Quasi-Monte Carlo (QMC) methods, such as the good lattice point method [8] and (t,m,s)-nets [9], are commonly used for constructing space-filling designs because they generate low-discrepancy sequences that ensure good coverage of the experimental domain. Another method for constructing space-filling designs is entropy criterion optimization [10]. By maximizing the entropy criterion [11,12], design points are ensured to spread points out [13,14,15], thus achieving maximum information gain and reducing uncertainty. Refs. [14,16] pointed out that, under the assumption of extremely weak correlation, maximum entropy designs tend to maximin distance designs [17], which are the most classical space-filling designs. Although space-filling designs, QMC methods, and entropy optimization may seem distinct at first glance, they are fundamentally interconnected through their shared goals of efficient sampling and maximizing the information obtained from a system. Therefore, the study of space-filling designs can be viewed as research on maximizing information gain from the experimental domain with a limited number of points, which falls at the intersection of statistics and information theory. In this paper, we primarily investigate the space-filling design method when branching and nested factors are involved.

The presence of branching and nested factors brings numerous challenges for generating the space-filling designs because different trials of an experiment can involve different factors, which are determined by the levels of branching factors. On the other hand, the nested factors are connected to branching factors, meaning that they are not independent. Moreover, such experiments commonly incorporate multiple types of factors, such as qualitative and quantitative factors. These characteristics make traditional space-filling design criteria unsuitable for evaluating designs with branching and nested factors. Naturally, the corresponding design strategies also become invalid. Recently, some studies have proposed space-filling criteria suitable for such experiments and provided corresponding design methods. Ref. [1] proposed the criterion from the perspectives of maximin distance. Unlike traditional criteria, such a criterion firstly assesses the sub-designs (including the nested and shared factors) under each level of each branching factor separately, secondly evaluates the design for shared factors, and then combines all of them to obtain the criteria for the entire design. Ref. [4] proposed a class of linear models suitable for experiments with branching and nested factors and used the D-optimality criterion to find the optimal designs. Ref. [5] proposed a uniformity criterion for such a design and claimed that compared with the criterion proposed by [1], this criterion allows all factors to be either qualitative or quantitative, and it imposes no restrictions on the number of levels for quantitative factors, making it applicable to a wider range of experiments.

However, the projection properties of these strategies are often ignored. In most experiments, only a small portion of factors involved are significant. Ref. [18] suggests that, if we only focus on the overall space-filling property of the design, it may result in poor projection uniformity in the dimensions of the significant factors, thereby reducing the efficiency of the experiment. Ref. [18] proposed a criterion for constructing maximum projection (Maxpro) designs. Optimal designs under this criterion can ensure space-filling properties in projections onto all subsets of factors. Ref. [19] extended this criterion to experiments involving both qualitative and quantitative factors. However, these criteria are not applicable to experiments with branching and nested factors.

In this paper, for experiments with branching and nested factors, we introduce a novel space-filling criterion by extending the Maxpro criterion in [19]. The paper is organized as follows. In Section 2, we provide some preliminaries relevant to this work. Section 3 reviews existing design criteria for experiments with branching and nested factors, pointing out their limitations. Section 4 introduces the novel criterion. Section 5 provides efficient methods for constructing optimal designs under the proposed criterion. Numerical comparisons are provided in Section 6 to demonstrate the effectiveness of the proposed methods. Section 7 concludes this paper.

## 2. Preliminaries

For a design D with *n* runs and *m* factors, where all factors are quantitative, plentiful criteria are proposed to find a “good” design. Ref. [20] suggested to choose designs with a minimal average of squared correlations between factors, defined as

(1) $$\begin{array}{c} \rho^{2}\left(D\right)=\frac{\sum_{i\neq j}\rho_{ij}^{2}}{m(m-1)}, \end{array}$$

where $\rho_{ij}$ is the linear correlation between the *i*th and *j*th columns. Ref. [16] proposed a criterion to construct the maximin Latin hypercube designs, given by

(2) $$\begin{array}{c} \phi_{\lambda }\left(D\right)={\left\{\sum_{i\neq j}d{\left(x_{i},x_{j}\right)}^{-\lambda }\right\}}^{1/\lambda }, \end{array}$$

where $d\left(x_{i},x_{j}\right)={\left(\sum_{k=1}^{m}{\left|x_{ik}-x_{jk}\right|}^{p}\right)}^{1/p}$ is the distance between the points $x_{i}$ and $x_{j}$ of the design D. Hereinafter, we set p=1. The design points of the resulting design optimized by (2) try to scatter evenly across the design space. Ref. [18] proposed the following performance measure

(3) $$\begin{array}{c} \psi \left(D\right)={\left\{\frac{1}{n(n-1)}\sum_{i\neq j}\frac{1}{\prod_{k=1}^{m}{\left(x_{ik}-x_{jk}\right)}^{2}}\right\}}^{1/m}. \end{array}$$

A Maxpro design minimizes the ψ value in (3), which aims to simultaneously optimize the space-filling properties of the design points across all possible subsets of factors.

On the other hand, some researchers focus on the design with multiple types of factors. Suppose that there are $p_{1}$ continuous quantitative factors, $p_{2}$ discrete quantitative factors, and $p_{3}$ qualitative factors, Ref. [19] proposed a new criterion to accommodate multiple types of factors,

(4) $$\begin{array}{c} \psi \left(D\right)={\left\{\frac{1}{n(n-1)}\psi^{*}\left(D\right)\right\}}^{1/(p_{1}+p_{2}+p_{3})}, \end{array}$$

where

$$\psi^{*}\left(D\right)=\sum_{i\neq j}\frac{1}{\prod_{k=1}^{p_{1}}{\left(x_{ik}-x_{jk}\right)}^{2}\prod_{k=p_{1}+1}^{p_{1}+p_{2}}{\left\{|x_{ik}-x_{jk}|+\frac{1}{q_{k}}\right\}}^{2}\prod_{k=p_{1}+p_{2}+1}^{p_{1}+p_{2}+p_{3}}{\left\{I\left(x_{ik}\neq x_{jk}\right)+\frac{1}{q_{k}}\right\}}^{2}},$$

$I(x_{ik}\neq x_{jk})$ is an indicator function, taking the value 1 if $x_{ik}$ is not equal to $x_{jk}$, and 0 otherwise. Moreover, $q_{k}$ is the number of levels in the *k*th column. Before calculating (4), each quantitative factor should be scaled into [0, 1]. Furthermore, Ref. [21] proposed the qualitative–quantitative discrepancy (QQD) to investigate the uniformity performance of the design with $m_{1}$ qualitative and $m_{2}$ quantitative factors, given by

$$\begin{array}{cc} {\mathrm{QQD}}^{2}\left(D\right)= & -\prod_{k=1}^{m_{1}}\left(\frac{5q_{k}+1}{4q_{k}}\right){\left(\frac{4}{3}\right)}^{m_{2}}+\frac{1}{n^{2}}\sum_{i,j=1}^{n}{\left(\frac{5}{4}\right)}^{m_{1}}{\left(\frac{6}{5}\right)}^{\delta_{ij}\left(D_{1}\right)}\times \\ & \prod_{k=m_{1}+1}^{m_{1}+m_{2}}\left(\frac{3}{2}-|x_{ik}-x_{jk}|+|x_{ik}-x_{jk}{\;|}^{2}\right), \end{array}$$

where $\delta_{ij}\left(D_{1}\right)$ represents the coincidence number between the *i*th and *j*th points of the qualitative sub-design $D_{1}$.

## 3. Existing Criteria and Limitations

Limited researches have been conducted on the experimental designs with branching and nested factors. There are two primary research directions, namely deterministic constructions [22,23,24] and the criteria. The existing conditions of the deterministic strategy are quite restrictive, while the criteria-based approach offers greater flexibility and adaptability to a variety of situations.

To the best of our knowledge, only two model-independent criteria have been customized for the designs including branching and nested factors. One is proposed by [1], by extending the maximin criterion (2), given by

(5) $$\begin{array}{c} \Phi_{\lambda }\left(D\right)={\left\{\sum_{i\neq j}{\left(\frac{t}{d_{S}\left(x_{i},x_{j}\right)}\right)}^{\lambda }+\sum_{l=1}^{r}\sum_{z=1}^{q_{l}}\sum_{i,j\in U_{l}^{z}}{\left(\frac{t+t_{l}}{d_{N}\left(x_{i},x_{j}\right)+d_{S}\left(x_{i},x_{j}\right)}\right)}^{\lambda }\right\}}^{1/\lambda }, \end{array}$$

where *t* and $t_{l}$ are the number of shared factors and nested factors corresponding to the *l*th branching factor, and $U_{l}^{z}$ gathers the row indices where the *l*th branching factor at level *z*; besides that, the subscripts *S* and *N* mean the projections of design points onto shared and nested factors, respectively. This is the first systemic measure to evaluate the goodness of a design with branching and nested factors. The design strategy suggested by [1] imposed a constraint on the branching factor, requiring it to be an orthogonal array. It also assumed that the nested and shared factors are quantitative with *n* distinct levels, making it only suitable for a computer experiment. From Formula (5), it is evident that the resulting design takes no account of the space-filling properties between the branching factor and the other factors; that is, such a criterion only considers the space-filling property among the nested and shared factors. Moreover, as mentioned in [19], the criterion in (2) pursues the space-fillingness in full dimensional space, whereas its projection properties in terms of subspaces for dimensions 2,⋯,m−1 are not ensured. The measure in (5) originates from (2); as a consequence, it shares the same shortcomings.

**Figure 1:**
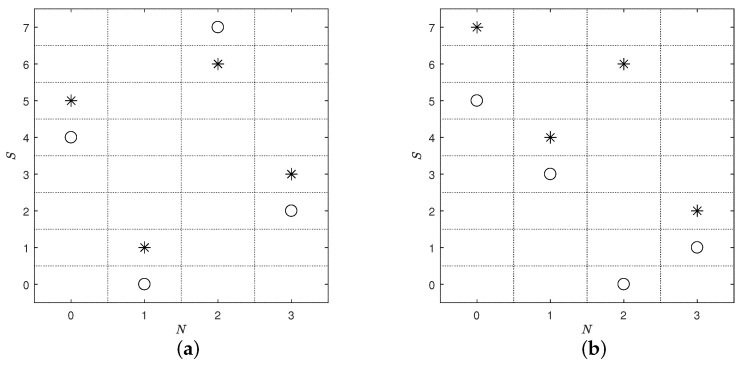
The points for designs in Table 1, where “*” corresponds to B=0 and “∘” corresponds B=1. (**a**) $D_{1}$; (**b**) $D_{2}$.

Ref. [5] tried to investigate the physical experiment systems with multiple types of factors, and introduced a criterion for experiments in the presence of nested factors from the perspective of uniformity

(6) $$\begin{array}{c} \Psi \left(D\right)=\frac{\mathrm{QQD}(D_{BS})}{2^{r+t}}+\sum_{l=1}^{r}\sum_{z=0}^{q_{l}-1}\frac{\mathrm{QQD}(D_{BNS}^{l,z})}{2^{r-1+t+t_{l}}}, \end{array}$$

where $D_{BS}$ is the sub-design that D projects on branching and shared factors; in addition, $D_{BNS}^{l,z}$ represents the sub-design consisting of all rows (consisting of the other branching, associated nested, and shared factor) within the *l*th branching factor taking level *z*. However, through our observation, the discrimination sensitivity of criterion (6) is relatively poor. Two illustrative designs with the same Ψ-value are given in Table 1, and their design points are shown in Figure 1. It is clear that the design points of Figure 1a are more space-filling than those of Figure 1b with respect to each level of the branching factor. However, they are considered to be equivalent under the criterion (6), which is inconsistent with the intuition. This indicates that the criterion in (6) fails to distinguish these two designs. On the other hand, although uniform designs perform well under uniformity criteria, their performance may not be satisfactory in terms of other space-filling properties, referring to Figure 1 of [18]. Consequently, the performance of the uniform designs with branching and nested factors in terms of other space-filling properties is still a concern. From the discussion above, a new criterion is required to deal with the experiments with multiple types of branching and nested factors.

## 4. Maxpro Criterion for Experiments with Multiple Types of Branching and Nested Factors

Throughout this paper, for the sake of clarity and without causing confusion, we will often use B, N, and S to represent the branching factor, nested factor, and shared factor, respectively. For simplicity, if there exist $m_{1}$ quantitative factors and $m_{2}$ qualitative factors, a modified criterion of (4) is given

(7) $$\begin{array}{c} \psi \left(D\right)={\left\{\sum_{i\neq j}\frac{1}{\prod_{k=1}^{m_{1}}{\left\{|x_{ik}-x_{jk}|+\frac{1}{q_{k}}\right\}}^{2}\prod_{k=m_{1}+1}^{m_{1}+m_{2}}{\left\{I\left(x_{ik}\neq x_{jk}\right)+\frac{1}{q_{k}}\right\}}^{2}}\right\}}^{1/(m_{1}+m_{2})}. \end{array}$$

More generally, let there be *m* factors, each of which can be either qualitative and quantitative. Let $\xi (x_{ik},x_{jk})$ be a binary function that takes value ${\left\{|x_{ik}-x_{jk}|+\frac{1}{q_{k}}\right\}}^{2}$ if the *k*th factor is quantitative and ${\left\{I\left(x_{ik}\neq x_{jk}\right)+\frac{1}{q_{k}}\right\}}^{2}$ otherwise. Then, (7) becomes

(8) $$\begin{array}{c} \psi \left(D\right)={\left\{\sum_{i\neq j}\frac{1}{\prod_{k=1}^{m}\xi \left(x_{ik},x_{jk}\right)}\right\}}^{1/m}. \end{array}$$

Now, we develop a new criterion for evaluating the goodness of designs for experiments including multiple types of branching and nested factors, by extending (8). Begin with a simple example where only one branching factor with $q_{1}$ levels exists. The Maxpro criterion for such a design can be defined as

$$\begin{array}{c} \Upsilon \left(D\right)=\psi \left(D_{BS}\right)+\sum_{z=0}^{q_{1}-1}\psi \left(D_{NS|b_{1}=z}\right), \end{array}$$

where $D_{NS|b_{1}=z}$ represents the sub-design of that design D projects onto nested and shared factors where the branching factor is at level *z*.

More generally, let there be *r* branching factors, and then the Maxpro criterion should be

(9) $$\begin{array}{c} \Upsilon \left(D\right)=\psi \left(D_{BS}\right)+\sum_{l=1}^{r}\sum_{z=0}^{q_{l}-1}\psi \left(D_{B_{\left(-l\right)}N^{l}S|b_{l}=z}\right), \end{array}$$

where $D_{B_{\left(-l\right)}N^{l}S|b_{l}=z}$ is the sub-design that consists of all the branching factors (except the *l*th one), the associated nested factor corresponding to the *l*th branching factors, and the shared factors, where the *l*th branching factor takes level *z*. Essentially, $D_{B_{\left(-l\right)}N^{l}S|b_{l}=z},l=1,\cdots ,r,z=0,\cdots ,q_{l}-1$ can be regarded as a series of sliced (or small) designs generated according to each level of each branching factor. The first part addresses the overall properties of the branching and shared space, while the second part of (9), related to $\sum_{l=1}^{r}q_{l}$ small designs involving branching, nested, and shared factors, examines the properties of these small designs at each level of every branching factor. The design with branching and nested factors corresponding to the minimal Υ value is called the optimal Maxpro BNS design, which takes into account the characteristics among the three kinds of factors. This criterion is based on the work of [19]; therefore, it inherits all its advantages and can also handle cases with branching and nested factors. The proposed criterion imposes no restrictions on run size and level, and is suitable for multiple types of factors.

**Figure 2:**
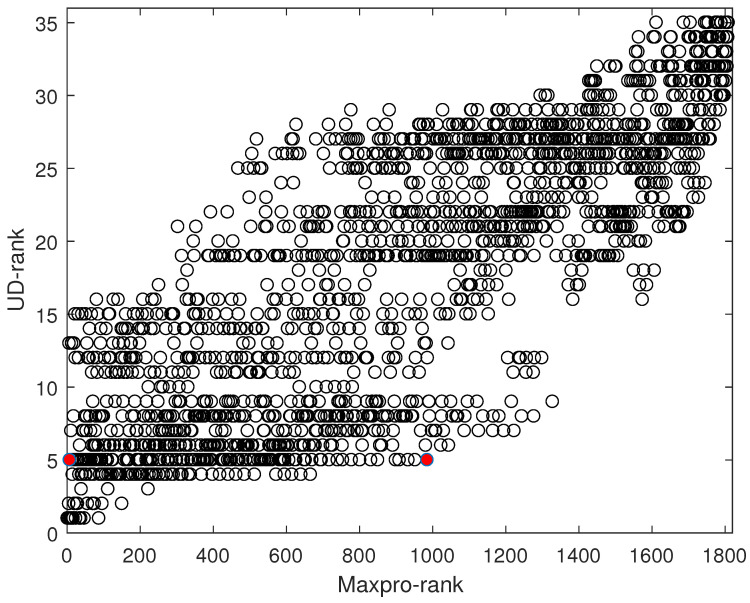
Maxpro rankings versus UD rankings, where the two designs marked by red color share the same UD ranking but different Maxpro rankings.

A similar structure of the objective criterion was proposed in [5]. The key difference is that, here, we focus on the Maxpro properties, whereas [5] concentrates on the uniformity. Both criteria are effective for searching optimal designs. However, there is no one-to-one relationship between these two criteria; in other words, the optimal designs under such two criteria can differ. To explain this point, consider all the eight-point designs with one two-level branching factor, one four-level nested factor, and one eight-level shared factor. There are a total of 8!= 40,320 non-isomorphic designs that cannot be obtained from each other through row and level permutations. These designs can be ranked in terms of the uniformity and the Maxpro criterion, (6) and (9), respectively, where under both criteria, the design ranked first is the corresponding best design. There are a total of 1891 combinations of ranking, with 1809 rankings on the Maxpro and 35 rankings on the UD, referring to Figure 2. It indicates the Maxpro criterion offers superior discrimination compared to the UD criterion. To elaborate on this, comparing the two designs colored by red in Figure 2, both of them are ranked 5th in terms of the UD criterion, but their rankings under Maxpro criterion are significantly different, being 7th and 985th, respectively. In fact, they are exactly the two designs in Table 1, where $D_{1}$ corresponds to the ranking seventh under the Maxpro criterion, and $D_{2}$ corresponds to another one. Despite both designs having the same UD value, the Maxpro values indicate that the design in Figure 1a is more desirable, which aligns with our intuition. On the other hand, it can be seen that the optimality of one criterion may not lead to the optimality of another. This issue becomes more severe as the number of factors and runs increases. As a result, the proposed criterion is able to distinguish the quality of designs involving branching and nested factors more efficiently.

## 5. Optimal Design Construction

### 5.1. Construction Method

Several stochastic algorithms can be utilized to construct the optimal designs, such as the threshold accepting method [7], simulated annealing algorithm [16], and stochastic evolutionary algorithm [25]. A standard procedure is as follows:

1. Start with a beginning design.
2. Develop a neighbor design of the current design by using the coordinate exchange algorithm.
3. Calculate the Υ-value for the neighbor design, and then decide whether to replace the current design or not.

Now, we give the method to generate the beginning design with branching and nested factors. Such a design consists of three parts. To spread the design points evenly on each dimension, for the branching and shared parts, U-type designs should be adopted, as well as the nested part corresponding to each level of the branching factors utilizing a smaller U-type design.

**Construction** **1.** *Beginning design:*

*1.*  *Randomly generate an n×r U-type design B to accommodate the branching factors.*
*2.*  *For level z of the lth branching factor, randomly generate a smaller $\left(n/q_{l}\right)\times t_{l}$ U-type design for $l=1,\cdots ,r;z=0,\cdots ,q_{l}-1$, say $N_{z}^{l}$, and then integrate them based on the levels of the branching factors to accommodate the nested factor $N^{l}$, which corresponds to the lth branching factor.*
*3.*  *Randomly generate an n×t U-type design S to arrange the nested factors.*
*4.*  *Let*
(10) $$\begin{array}{c} D=(B,N^{1},\cdots ,N^{r},S), \end{array}$$
*which consists of r branching factors, $t_{l}$ factors nested into the lth branching factors, and t shared factors. Thus, there are $r+\sum_{l=1}^{r}t_{l}+t$ factors in total.*

For the constructed design in (10), assume the level of *k*th factor is $q_{k},k=1,\cdots ,r+\sum_{l=1}^{r}t_{l}+t$. We give an example to illustrate each step of Construction 1.

**Example** **1.** *Let $n=8,r=1,t_{1}=1,t=1,q_{1}=2,q_{2}=4,q_{3}=8$, and then a possible outcome from Steps 1–3 can be*

$$\begin{array}{c} \begin{array}{ccccc} B=\left(\begin{array}{c} 0\\ 1\\ 0\\ 0\\ 1\\ 1\\ 0\\ 1 \end{array}\right), & N_{0}^{1}=\left(\begin{array}{c} 0\\ 1\\ 2\\ 3 \end{array}\right), & N_{1}^{1}=\left(\begin{array}{c} 3\\ 2\\ 0\\ 1 \end{array}\right), & N^{1}=\left(\begin{array}{c} 0\\ 3\\ 1\\ 2\\ 2\\ 0\\ 3\\ 1 \end{array}\right), & S=\left(\begin{array}{c} 2\\ 6\\ 0\\ 5\\ 7\\ 3\\ 4\\ 1 \end{array}\right), \end{array} \end{array}$$

*Note that the elements of $N^{1}$ coming from $N_{0}^{1}$ are bold, corresponding to the rows where the branching factor is at level 0. In Step 4, generate the initial design*

$$D=\left(B,N^{1},S\right)=\left(\begin{array}{ccc} 0 & 0 & 2\\ 1 & 3 & 6\\ 0 & 1 & 0\\ 0 & 2 & 5\\ 1 & 2 & 7\\ 1 & 0 & 3\\ 0 & 3 & 4\\ 1 & 1 & 1 \end{array}\right)$$

For simplicity, some sets are introduced to collect the column indices where some sub-designs are located. Let $\delta_{B}=\left\{1,\cdots ,r\right\}$, that is, column $i\in \delta_{B}$ of design D in (10) is a branching factor. In the same fashion, let $\delta_{N^{l}}=\left\{r+1+\sum_{p=1}^{l}t_{p-1},\cdots ,r+\sum_{p=1}^{l}t_{p}\right\}$ for l=1,⋯,r, and $\delta_{S}=\left\{r+1+\sum_{l=1}^{r}t_{l},\cdots ,r+\sum_{l=1}^{r}t_{l}+t\right\}$, corresponding to the column indices where the nested factors are for the *l*th branching factors and the shared factors, respectively. More generally, let $\delta_{BS}=\delta_{B}\cup \delta_{S}$ and $\delta_{B_{\left(-l\right)}N^{l}S}=\delta_{B}\cup \delta_{N^{l}}\cup \delta_{S}\setminus \left\{l\right\}$, such as, for D in Example 1, $\delta_{BS}=\left\{1,3\right\}$ and $\delta_{B_{\left(-1\right)}N^{1}S}=\left\{2,3\right\}$.

To generate a neighbor design that maintains the branching–nested relationship, we provide the following construction by modifying the coordinate exchange algorithm.

**Construction** **2.** *Neighbor design:*

*1.*  *Draw a random column number u from $\{1,\cdots ,r+t+\sum_{l=1}^{r}t_{l}\}.$*
*2.*  *If $u\in \delta_{B}$, the selected factor is a branching factor, then exchange two randomly locations ($w_{1},w_{2}$) within the uth column, and also exchange the $w_{1}$ and $w_{2}$ rows of the $N^{u}$. There are $2+2t_{u}$ changed elements in total.*
*3.*  *If $u\in \delta_{N^{l}},l=1,\cdots ,r$, the selected factor is a nested factor nested into the lth branching factor, then exchange two randomly locations ($w_{1},w_{2}$) of the uth column where the associated branching factor has the same level.*
*4.*  *If $u\in \delta_{S}$, the selected factor is a shared factor, then exchange two randomly locations ($w_{1},w_{2}$) of uth column.*

**Example** **2.** *Consider neighbor designs of the current D in Example 1.*

*(a)*  *If u=1, turn to Step 2, and suppose $w_{1}=1,w_{2}=2$; then, we can obtain $D_{a}$. Here, if we only swap the first and second elements of the branching factor and ignore the associated nested factor, the branching–nested structure will be disrupted.*
*(b)*  *If u=2, go to Step 3. Two selected locations should correspond to the same level of the branching factor (such as level 0), i.e., $w_{1},w_{2}\in \left\{1,3,4,7\right\}$. Let $w_{1}=1,w_{2}=7$, and then the resulting design is $D_{b}$. In this situation, if two elements come from different levels of the branching factor, then the branching–nested structure should also be broken.*
*(c)*  *If u=3, proceed to Step 4, and assume $w_{1}=2,w_{2}=3$; then, we have $D_{c}$.*

$$D_{a}=\left(\begin{array}{ccc} 1 & 3 & 2\\ 0 & 0 & 6\\ 0 & 1 & 0\\ 0 & 2 & 5\\ 1 & 2 & 7\\ 1 & 0 & 3\\ 0 & 3 & 4\\ 1 & 1 & 1 \end{array}\right),\;D_{b}=\left(\begin{array}{ccc} 0 & 3 & 2\\ 1 & 3 & 6\\ 0 & 1 & 0\\ 0 & 2 & 5\\ 1 & 2 & 7\\ 1 & 0 & 3\\ 0 & 0 & 4\\ 1 & 1 & 1 \end{array}\right),\;D_{c}=\left(\begin{array}{ccc} 0 & 0 & 2\\ 1 & 3 & 0\\ 0 & 1 & 6\\ 0 & 2 & 5\\ 1 & 2 & 7\\ 1 & 0 & 3\\ 0 & 3 & 4\\ 1 & 1 & 1 \end{array}\right).$$

*The exchanged elements in $D_{a},D_{b},D_{c}$ are in bold.*

### 5.2. Iterative Formulas

From Construction 2, the exchanging strategies depend heavily on the kinds of the selected factor. In each iteration (neighbor design), only a few elements have been changed. Through our observations, the value of (9) relies on $1+\sum_{l=1}^{r}q_{l}$ sud-designs, many of which remain unchanged after one iteration. Therefore, the criterion can be efficiently updated without recalculating all parts of (9) at each iteration. The neighbor design of D is denoted by $D^{*}$. It is feasible to skillfully compute the new $\Upsilon (D^{*})$ by leveraging the previous $\psi (D_{BS})$ and $\psi (D_{B_{\left(-l\right)}N^{l}S|b_{l}=z})$. We have

(11) $$\begin{array}{c} \Upsilon \left(D^{*}\right)=\psi \left(D_{BS}^{*}\right)+\sum_{l=1}^{r}\sum_{z=0}^{q_{l}-1}\psi \left(D_{B_{\left(-l\right)}N^{l}S|b_{l}=z}^{*}\right). \end{array}$$

The computational complexity of (11) consists of two parts. The calculation of the first and second parts will take O$\left(n^{2}\left(r+t\right)\right)$ and $\sum_{l=1}^{r}$O$\left(\left(n^{2}/q_{l}\right)\left(r-1+t_{l}+t\right)\right)$, respectively. In the rest of this section, three possible neighbors from Steps 2, 3, and 4 of Construction 2 are discussed, respectively, and the iterative formulas for them are derived separately.

(a) If $u\in \delta_{B}$, since the branching factor determines the manner of partitioning, it becomes quite complex, and thus, the iterative formula for the second part of (11) is not considered. The first part of (11) can be updated iteratively. Let $h_{BS}\left(x_{i},x_{j}\right)=\prod_{k\in \delta_{BS}}\xi \left(x_{ik},x_{jk}\right)$, and $x_{i}$ and $x_{j}$ correspond to the *i*th and *j*th rows of design D. Note that $D_{BS}^{*}$ is obtained by interchanging the $w_{1}$ and $w_{2}$ positions of the *u*th column of $D_{BS}$; then, for 1≤i≤n and $i\neq w_{1},w_{2}$, we have
  $$\begin{array}{c} h_{BS}\left(x_{i},x_{w_{1}}^{*}\right)=h_{BS}\left(x_{i},x_{w_{1}}\right)\frac{\xi (x_{iu},x_{w_{2}u})}{\xi (x_{iu},x_{w_{1}u})} \end{array}$$
  and
  $$\begin{array}{c} h_{BS}\left(x_{i},x_{w_{2}}^{*}\right)=h_{BS}\left(x_{i},x_{w_{2}}\right)\frac{\xi (x_{iu},x_{w_{1}u})}{\xi (x_{iu},x_{w_{2}u})}, \end{array}$$
  where $x_{w_{1}}^{*}$ and $x_{w_{2}}^{*}$ are the $w_{1}$th and $w_{2}$th rows of $D_{BS}^{*}$. It can be easily derived that
  (12) $$\begin{array}{cc} {\left[\psi \left(D_{BS}^{*}\right)\right]}^{r+t}= & {\left[\psi \left(D_{BS}\right)\right]}^{r+t}+2\sum_{i(\neq w1,w2)=1}^{n}\left(\frac{1}{h_{BS}\left(x_{i},x_{w_{1}}^{*}\right)}-\frac{1}{h_{BS}\left(x_{i},x_{w_{1}}\right)}\right)\\ \; & +2\sum_{i(\neq w1,w2)=1}^{n}\left(\frac{1}{h_{BS}\left(x_{i},x_{w_{2}}^{*}\right)}-\frac{1}{h_{BS}\left(x_{i},x_{w_{2}}\right)}\right), \end{array}$$
  for which the computational complexity is O(n).
(b) If $u\in \delta_{N^{l}},l=1,\cdots ,r$, assume the selected locations $\left(w_{1},w_{2}\right)$ in the *u*th column correspond to level $i_{0}$ of the $l_{0}$th branching factor. Clearly, the sub-design for branching and shared factors remains unchanged, and thus, the value for the first part of (11) does not change; that is,
  $$\begin{array}{c} {\left[\psi \left(D_{BS}^{*}\right)\right]}^{r+t}={\left[\psi \left(D_{BS}\right)\right]}^{r+t}. \end{array}$$
  As for the second part of (11), among the $\sum_{l=1}^{r}q_{l}$ terms involved, only one of them is subject to change, as follows,
  $$\begin{array}{cc} \; & \psi {\left(D_{B_{\left(-l\right)}N^{l}S|b_{l}=z}^{*}\right)}^{r-1+t+t_{l}}\\ \; & =\left\{\begin{array}{cc} \psi {\left(D_{B_{\left(-l\right)}N^{l}S|b_{l}=z}\right)}^{r-1+t+t_{l}}\\ +2\sum_{i\in I(\neq w1,w2)}\left(\frac{1}{h_{B_{\left(-l\right)}N^{l}S}\left(x_{i},x_{w_{1}}^{*}\right)}-\frac{1}{h_{B_{\left(-l\right)}N^{l}S}\left(x_{i},x_{w_{1}}\right)}\right)\\ +2\sum_{i\in I(\neq w1,w2)}\left(\frac{1}{h_{B_{\left(-l\right)}N^{l}S}\left(x_{i},x_{w_{2}}^{*}\right)}-\frac{1}{h_{B_{\left(-l\right)}N^{l}S}\left(x_{i},x_{w_{2}}\right)}\right), & \mathrm{if}\;l=l_{0}\;\mathrm{and}\;z=z_{0},\\ \psi {\left(D_{B_{\left(-l\right)}N^{l}S|b_{l}=z}\right)}^{r-1+t+t_{l}}, & \mathrm{otherwise}, \end{array}\right. \end{array}$$
  where *I* is a set, collecting row indices where the $l_{0}$th branching factor takes level $z_{0}$. In addition, $h_{B_{\left(-l\right)}N^{l}S}\left(\cdot ,\cdot \right)$ can be similarly defined as $h_{BS}\left(\cdot ,\cdot \right)$. The computation will take O$\left(n/q_{l_{0}}\right)$, if $l=l_{0}$ as well as $z=z_{0}$, and 0 otherwise.
(c) If $u\in \delta_{S}$, ${\left[\psi \left(D_{BS}^{*}\right)\right]}^{r+t}$ can be calculated by (12). In addition, depending on the levels of branching factors at $w_{1}$ and $w_{2}$ positions, it can be further divided into the following two cases:
  (i) The $w_{1}$ and $w_{2}$ runs of the *l*th branching factor have the same level, say level *c*, denoting the corresponding run indices such that the *l*th branching factor takes level *c* as $I_{c}^{l}$, then
    $$\begin{array}{cc} & \psi {\left(D_{B_{\left(-l\right)}N^{l}S|b_{l}=z}^{*}\right)}^{r-1+t+t_{l}}\\ \; & =\left\{\begin{array}{cc} \psi {\left(D_{B_{\left(-l\right)}N^{l}S|b_{l}=z}\right)}^{r-1+t+t_{l}}, & \mathrm{if}\;z\neq c,\\ \psi {\left(D_{B_{\left(-l\right)}N^{l}S|b_{l}=z}\right)}^{r-1+t+t_{l}}\\ +2\sum_{i\in I_{c}^{l}\left(\neq w1,w2\right)}\left(\frac{1}{h_{B_{\left(-l\right)}N^{l}S}\left(x_{i},x_{w_{1}}^{*}\right)}-\frac{1}{h_{B_{\left(-l\right)}N^{l}S}\left(x_{i},x_{w_{1}}\right)}\right)\\ +2\sum_{i\in I_{c}^{l}\left(\neq w1,w2\right)}\left(\frac{1}{h_{B_{\left(-l\right)}N^{l}S}\left(x_{i},x_{w_{2}}^{*}\right)}-\frac{1}{h_{B_{\left(-l\right)}N^{l}S}\left(x_{i},x_{w_{2}}\right)}\right), & \mathrm{if}\;z=c. \end{array}\right. \end{array}$$
  (ii) The $w_{1}$ and $w_{2}$ runs of the *l*th branching factor take two distinct levels, say level $c,c^{\prime }$, respectively. Similarly, define $I_{c}^{l},I_{c^{\prime }}^{l}$, then
    $$\begin{array}{cc} & \psi {\left(D_{B_{\left(-l\right)}N^{l}S|b_{l}=z}^{*}\right)}^{r-1+t+t_{l}}\\ \; & =\left\{\begin{array}{cc} \psi {\left(D_{B_{\left(-l\right)}N^{l}S|b_{l}=z}\right)}^{r-1+t+t_{l}}, & \mathrm{if}\;z\neq c,c^{\prime },\\ \;\psi {\left(D_{B_{\left(-l\right)}N^{l}S|b_{l}=z}\right)}^{r-1+t+t_{l}}\\ +2\sum_{i\in I_{c}^{l}\left(\neq w1,w2\right)}\left(\frac{1}{h_{B_{\left(-l\right)}N^{l}S}\left(x_{i},x_{w_{1}}^{*}\right)}-\frac{1}{h_{B_{\left(-l\right)}N^{l}S}\left(x_{i},x_{w_{1}}\right)}\right), & \mathrm{if}\;z=c,\\ \;\psi {\left(D_{B_{\left(-l\right)}N^{l}S|b_{l}=z}\right)}^{r-1+t+t_{l}}\\ +2\sum_{i\in I_{c^{\prime }}^{l}\left(\neq w1,w2\right)}\left(\frac{1}{h_{B_{\left(-l\right)}N^{l}S}\left(x_{i},x_{w_{2}}^{*}\right)}-\frac{1}{h_{B_{\left(-l\right)}N^{l}S}\left(x_{i},x_{w_{2}}\right)}\right), & \mathrm{if}\;z=c^{\prime }. \end{array}\right. \end{array}$$
  In the same fashion, the computational complexity can be given.

Let $k_{l}=r-1+t+t_{l}$. Overall, the computational complexity of (11) by using the original method is O$\left(n^{2}\left(r+t\right)+\sum_{l=1}^{r}\left(n^{2}/q_{l}\right)k_{l}\right)$. Theorem 1 summarizes the complexities of the iterative method for three different cases.

**Theorem** **1.** *The computational complexities for* (11) *by using the iterative method are as follows.*

*(a)*  *If a branching factor is selected, the computational complexity is O$\left(n+\sum_{l=1}^{r}\left(n^{2}/q_{l}\right)k_{l}\right)$.*
*(b)*  *If a nested factor associated with the $l_{0}$th branching factor is selected, the computational complexity is O$\left(n/q_{l_{0}}\right)$.*
*(c)*  *If a shared factor is selected, the computational complexity is O$\left(n+\sum_{l=1}^{r}\left(n/q_{l}\right)\right)$.*

Assume that each factor has an equal probability of being chosen, then from Step 1 of Construction 2, the probabilities of the three kinds of factors, i.e., branching, nested, and shared factors, being selected in each iteration are $r/(r+t+\sum_{l=1}^{r}t_{l})$, $\sum_{l=1}^{r}t_{l}/\left(r+t+\sum_{l=1}^{r}t_{l}\right)$, and $t/(r+t+\sum_{l=1}^{r}t_{l})$, respectively. From Theorem 1, it can be found that the computational complexity has been significantly reduced for each iteration.

## 6. Numerical Comparisons

In this section, we examine the performances of the low-dimensional projections for the optimal designs with branching and nested factors (for short, BNS designs) under (9), (5), (6), and the random design, (referred to as “Maxpro BNS”, “Hung BNS”, “UD BNS”, and “Random BNS”, respectively), across three criteria: (a) the maximin design criterion in (2), (b) the average correlation in (1), and (c) the Maxpro distance criterion in (3), denoted by “Mm”, “Ave-corr”, and “Maxpro”, respectively. For all the criteria, the smaller the better.

**Example** **3.** *Let there be one two-level branching factor, one twenty-level factor nested in the branching factor, and seven forty-level shared factors with forty points. To make a comparison, we have to assume that the branching factor is qualitative, while the rest of the factors are quantitative because Hung’s method has such limitations.*

**Figure 3:**
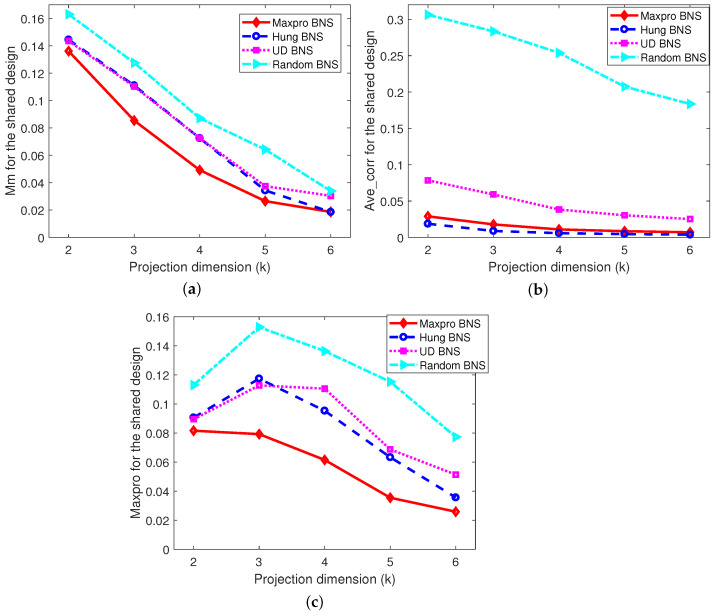
The comparisons of projection designs under “Mm”, “Ave_corr”, and “Maxpro”. (**a**) Mm; (**b**) Ave_corr; (**c**) Maxpro.

**Figure 4:**
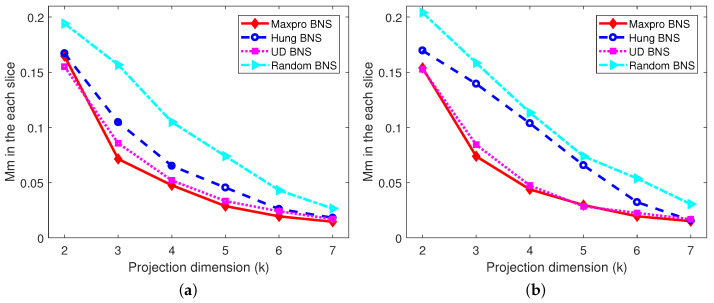
Comparisons of projection designs under “Mm”. (**a**) When B=0. (**b**) When B=1.

**Figure 5:**
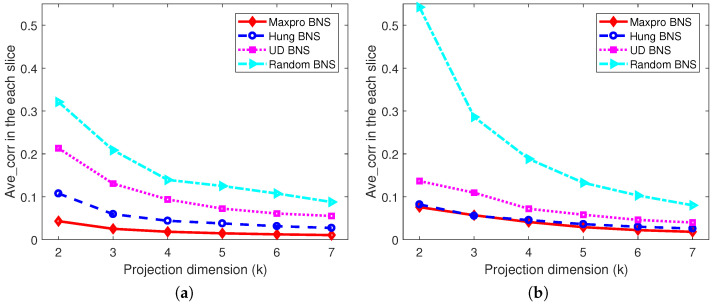
Comparisons of projection designs under “Ave_corr”. (**a**) When B=0. (**b**) When B=1.

**Figure 6:**
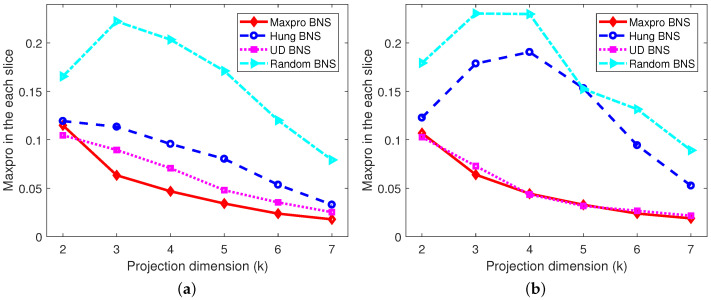
Comparisons of projection designs under “Maxpro”. (**a**) When B=0. (**b**) When B=1.

*The projected designs of the shared parts onto 2≤k≤6 dimensions, as well as the projected designs of the nested and shared parts onto 2≤k≤7 dimensions corresponding to each level of the branching factor are investigated, respectively. Only the worst cases are presented, for example, in Figure 3a, there are a total of 72=21 possible two-dimensional projected designs for the shared designs of the Maxpro BNS design, and the “Mm” value for the worst one among them is 0.1362. Figure 3 shows that the proposed Maxpro BNS design is the best under “Mm” and “Maxpro”, and performs well in terms of “Ave-corr”, just behind the Hung BNS design. As expected, the random BNS design is the worst under all the criteria. In addition, after partitioning the nested and shared factors into slices by the levels of the branching factor, from Figure 4, Figure 5 and Figure 6, it can be easily found that Maxpro BNS behaves substantially better with respect to “Mm”, “Ave_corr”, and “Maxpro”.*

To show that the proposed Maxpro BNS design can achieve better grid stratification than a UD BNS design, another simple example is presented.

**Figure 7:**
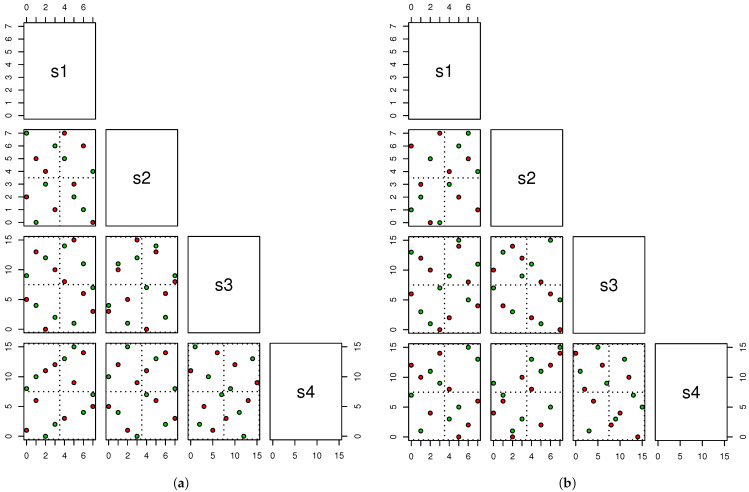
Scatter points of bivariate projections for $D_{1}$ and $D_{2}$ in Example 4. The red color corresponds to level 0 of the branching factor, while the green color corresponds to level 1 of the branching factor. (**a**) $D_{1}$; (**b**) $D_{2}$.

**Example** **4.** *Consider a sixteen-run design with one two-level qualitative branching factor, one two-level qualitative factor nested within it, and additionally, two eight-level quantitative and two sixteen-level quantitative shared factors (i.e., $r=1,t_{1}=1,t=4,q_{1}=q_{2}=2,q_{3}=q_{4}=8,q_{5}=q_{6}=16$). The optimal designs under Maxpro BNS and UD BNS criterion are listed in Table 2, denoted by $D_{1}$ and $D_{2}$, respectively. Scatter points of bivariate projection of $D_{1}$ and $D_{2}$ are shown in Figure 7. Note that the two designs are generated by stochastic methods without utilizing any specific structure. Surprisingly, the points of the Maxpro BNS design appear to achieve stratification on grids like the OA-based structure. Focus on Figure 7a, with exactly four points in each of the 2×2 grids for any two variables, as well as exactly two red/green points in each of the 2×2 grids. However, as depicted in Figure 7b, it is evident that $D_{2}$ cannot achieve such properties.*

## 7. Discussion

In this paper, we have introduced a novel criterion to quantify the performance of the designs with the branching and nested factors based on the maximum projection principle. We have developed a comprehensive framework for constructing optimal designs under this criterion and demonstrated its advantages through numerical comparisons, particularly in terms of maintaining superior space-filling properties across projections to nearly all subsets of factors. To enhance the efficiency of design optimization, we provide the iterative formulas that significantly reduce the computational complexity involved in the design optimization process.

There are some future research directions from this paper. One significant direction is the theoretical construction of optimal designs under the proposed criterion. There is potential to derive closed-form solutions for optimal designs under certain conditions. Such theoretical results will enhance the efficiency of design construction in practical applications. Another is to verify the performance of Maxpro BNS designs in various modeling contexts. For instance, studying the behavior of Maxpro designs in polynomial regression models or Gaussian process (GP) models. This study could provide valuable insights into their effectiveness in capturing complex relationships in experimental data, which helps to further validate the applicability of the Maxpro BNS designs.

## Figures and Tables

**Table 1:** Two designs with Ψ=0.023.

Run	$D_{1}$			$D_{2}$		
	B	N	S	B	N	S
1	0	0	5	0	0	7
2	0	1	1	0	1	4
3	0	2	6	0	2	6
4	0	3	3	0	3	2
5	1	0	4	1	0	5
6	1	1	0	1	1	3
7	1	2	7	1	2	0
8	1	3	2	1	3	1

**Table 2:** The Maxpro BNS design $D_{1}$ and the UD BNS design $D_{2}$.

Run	$D_{1}$						$D_{2}$					
	B	N	$S_{1}$	$S_{2}$	$S_{3}$	$S_{4}$	B	N	$S_{1}$	$S_{2}$	$S_{3}$	$S_{4}$
1	0	0	3	1	10	12	0	0	4	4	2	8
2	0	0	7	0	3	5	0	0	0	6	6	12
3	0	0	6	6	6	14	0	0	5	2	14	0
4	0	0	1	5	13	6	0	0	2	0	10	4
5	0	1	4	7	8	3	0	1	6	5	8	2
6	0	1	5	3	15	9	0	1	1	3	12	10
7	0	1	2	4	0	11	0	1	3	7	0	14
8	0	1	0	2	5	1	0	1	7	1	4	6
9	1	0	4	5	14	13	1	0	7	4	11	13
10	1	0	3	6	2	2	1	0	5	6	15	5
11	1	0	6	1	11	4	1	0	1	2	3	1
12	1	0	0	7	9	8	1	0	3	0	7	9
13	1	1	1	0	4	10	1	1	0	1	13	7
14	1	1	2	3	12	0	1	1	6	7	5	15
15	1	1	5	2	1	15	1	1	2	5	1	11
16	1	1	7	4	7	7	1	1	4	3	9	3

## Data Availability

The data presented in this study are available on request from the corresponding author.
